# Association of Longitudinal Changes in Cerebrospinal Fluid Total Tau and Phosphorylated Tau 181 and Brain Atrophy With Disease Progression in Patients With Alzheimer Disease

**DOI:** 10.1001/jamanetworkopen.2019.17126

**Published:** 2019-12-11

**Authors:** Jorge J. Llibre-Guerra, Yan Li, Suzanne E. Schindler, Brian A. Gordon, Anne M. Fagan, John C. Morris, Tammie L. S. Benzinger, Jason Hassenstab, Guoqiao Wang, Ricardo Allegri, Sarah B. Berman, Jasmeer Chhatwal, Martin R. Farlow, David M. Holtzman, Mathias Jucker, Johannes Levin, James M. Noble, Stephen Salloway, Peter Schofield, Celeste Karch, Nick C. Fox, Chengjie Xiong, Randall J. Bateman, Eric McDade

**Affiliations:** 1 Department of Neurology, Washington University in St Louis, St Louis, Missouri; 2 Department of Biostatistics, Washington University in St Louis, St Louis, Missouri; 3 Department of Radiology, Washington University in St Louis, St Louis, Missouri; 4 Department of Pathology and Immunology, Washington University School of Medicine in St Louis, St Louis, Missouri; 5 Hope Center for Neurological Disorders, St Louis, Missouri; 6 Knight Alzheimer’s Disease Research Center, St Louis, Missouri; 7 Hertie Institute for Clinical Brain Research, Department of Cellular Neurology, University of Tübingen, Tübingen, Germany; 8 Department of Cognitive Neurology, Institute for Neurological Research Fleni, Buenos Aires, Argentina; 9 Department of Neurology, University of Pittsburgh, Pittsburgh, Pennsylvania; 10 Massachusetts General Hospital, Harvard Medical School, Boston; 11 Neuroscience Center, Indiana University, Bloomington; 12 DZNE-German Center for Neurodegenerative Diseases, Tübingen, Tübingen, Germany; 13 Department of Neurology, Ludwig-Maximilians-University, Munich, Germany; 14 DZNE-German Center for Neurodegenerative Diseases, Munich, Munich, Germany; 15 SyNergy, Munich Cluster for Systems Neurology, Munich, Germany; 16 Taub Institute for Research on Alzheimer’s Disease, Aging Brain G.H. Sergievsky Center, Department of Neurology, Columbia University Medical Center, New York, New York; 17 Memory & Aging Program, Butler Hospital, Brown University, Providence, Rhode Island; 18 Neuroscience Research Australia, Randwick, Sydney, New South Wales, Australia; 19 School of Medical Sciences, UNSW Sydney, Sydney, New South Wales, Australia; 20 Department of Psychiatry, Washington University in St Louis, St Louis, Missouri; 21 Dementia Research Centre, University College London, London, United Kingdom

## Abstract

**Importance:**

The amyloid/tau/neurodegeneration (A/T/N) framework uses cerebrospinal fluid (CSF) levels of total tau (tTau) as a marker of neurodegeneration and CSF levels of phosphorylated tau 181 (pTau181) as a marker of tau tangles. However, it is unclear whether CSF levels of tTau and pTau181 have similar or different trajectories over the course of Alzheimer disease.

**Objectives:**

To examine the rates of change in CSF levels of tTau and pTau181 across the Alzheimer disease course and how the rates of change are associated with brain atrophy as measured by magnetic resonance imaging.

**Design, Setting, and Participants:**

This cohort study was set in tertiary research clinics. Each participant was a member of a pedigree with a known mutation for dominantly inherited Alzheimer disease. Participants were divided into 3 groups on the basis of the presence of a mutation and their Clinical Dementia Rating score. Data analysis was performed in June 2019.

**Main Outcomes and Measures:**

Rates of change of CSF tTau and pTau181 levels and their association with the rate of change of brain volume.

**Results:**

Data from 465 participants (283 mutation carriers and 182 noncarriers) were analyzed. The mean (SD) age of the cohort was 37.8 (11.3) years, and 262 (56.3%) were women. The mean (SD) follow-up duration was 2.7 (1.5) years. Two or more longitudinal CSF and magnetic resonance imaging assessments were available for 160 and 247 participants, respectively. Sixty-five percent of mutation carriers (183) did not have symptoms at baseline (Clinical Dementia Rating score, 0). For mutation carriers, the annual rates of change for CSF tTau and pTau181 became significantly different from 0 approximately 10 years before the estimated year of onset (mean [SE] rates of change, 5.5 [2.8] for tTau [*P* = .05] and 0.7 [0.3] for pTau 181 [*P* = .04]) and 15 years before onset (mean [SE] rates of change, 5.4 [3.9] for tTau [*P* = .17] and 1.1 [0.5] for pTau181 [*P* = .03]), respectively. The rate of change of pTau181 was positive and increased at the early stages of the disease, showing a positive rate of change starting at 15 estimated years before onset until 5 estimated years before onset (mean [SE], 0.4 [0.3]), followed by a positive but decreasing rate of change at year 0 (mean [SE], 0.1 [0.3]) and then negative rates of change at 5 years (mean [SE], −0.3 [0.4]) and 10 years (mean [SE], −0.6 [0.6]) after symptom onset. In individuals without symptoms (Clinical Dementia Rating score, 0), the rates of change of CSF tTau and pTau181 were negatively associated with brain atrophy (high rates of change in CSF measures were associated with low rates of change in brain volume in asymptomatic stages). After symptom onset (Clinical Dementia Rating score, >0), an increased rate of brain atrophy was not associated with rates of change of levels of both CSF tTau and pTau181.

**Conclusions and Relevance:**

These findings suggest that CSF tTau and pTau181 may have different associations with brain atrophy across the disease time course. These results have implications for understanding the dynamics of disease pathobiology and interpreting neuronal injury biomarker concentrations in response to Alzheimer disease progression and disease-modifying therapies.

## Key Points

**Question:**

How do different proposed measurements of tau brain pathologic abnormalities (ie, levels of phosphorylated tau 181 in the cerebrospinal fluid [CSF]) and neurodegeneration (ie, total tau levels in the CSF and brain atrophy) change over the course of Alzheimer disease?

**Findings:**

This cohort study of 465 participants with dominantly inherited Alzheimer disease found that the rates of change for CSF levels of total tau and phosphorylated tau 181 had a different pattern across the course of Alzheimer disease. The association between the rates of change of CSF levels of total tau and phosphoryated tau 181 and brain atrophy varied by disease stage.

**Meaning:**

These results may provide a better understanding of the dynamics of Alzheimer disease and have important implications as trials targeting tau brain pathologic abnormalities move forward.

## Teaser

This cohort study of participants with dominantly inherited Alzheimer disease examines the rates of change in cerebrospinal fluid levels of total tau (tTau) and phosphorylated tau 181 (pTau181) across the disease course and how the rates of change are associated with brain atrophy.

## Introduction

The neuropathologic hallmarks of Alzheimer disease (AD) are the presence of neuritic amyloid plaques, primarily of the amyloid-β peptide, and the intraneuronal accumulation of neurofibrillary tangles (NFTs) and neuropil threads composed of hyperphosphorylated, aggregated tau protein.^1,2^ Our understanding of AD has evolved substantially over the past 2 decades, with neuroimaging and fluid biomarkers allowing for early detection of AD-related pathologic abnormalities.^3,4,5^ In fewer than 1% of patients, AD is caused by autosomal dominant mutations in either the presenilin 1, presenilin 2, or amyloid precursor protein genes. Dominantly inherited AD (DIAD) is considered clinically similar to sporadic AD except for a younger age at onset (AAO).^6^ Mutation carriers (MCs) have a somewhat predictable age at AD symptom onset.^7^

Biomarker studies^6,8^ have contributed to hypothesized trajectories of fluid and imaging biomarker changes that occur over the course of the disease, from the preclinical phase to the end stages characterized by advanced dementia. However, models of biomarker change in AD have been based mostly on cross-sectional data.^6,9,10^ More recently, many models have classified biomarkers according to their proposed association with the biological underpinnings of the disease; the recent amyloid/tau/neurodegeneration (A/T/N) framework^11^ was developed to provide a more biological rationale to the classification of the disease. Phosphorylated tau 181 (pTau181) in the cerebrospinal fluid (CSF) has been suggested to represent NFT pathologic abnormalities, whereas total tau (tTau) in the CSF is thought to be a marker of neurodegeneration that is passively released with cell death or injury.^8,12^ Given these putative mechanisms, it might be expected that levels of both tTau and pTau181 would continue to become more abnormal with disease progression, as NFT pathologic abnormalities increase and neurodegeneration accelerates. However, recent longitudinal studies^13,14,15,16,17^ from the Dominantly Inherited Alzheimer Network (DIAN) and Alzheimer’s Disease Neuroimaging Initiative (ADNI) cohorts have challenged the linear model from previous cross-sectional studies,^3,9,18^ which have consistently found higher CSF levels of tTau and pTau181 as the disease progresses.

These findings highlight a need for accurate determination of the evolution of longitudinal changes in CSF levels of tTau and pTau181 and their association with disease progression. In the present study, we assessed the longitudinal pattern of changes in CSF levels of tTau and pTau181 and their association with brain atrophy as measured by magnetic resonance imaging (MRI). We hypothesized that if CSF tTau and pTau181 were passively released with neurodegeneration, they should be associated with MRI measures of neurodegeneration (eg, rate of atrophy). To evaluate this hypothesis, we used a well-characterized cohort with DIAD from the DIAN study.^19^

## Methods

All participants were recruited as part of the DIAN study.^20^ Participants provided written informed consent or assent with proxy consent. The institutional review boards for each of the participating DIAN sites approved all aspects of the study. This study follows the Strengthening the Reporting of Observational Studies in Epidemiology (STROBE) reporting guideline.

For our analysis, 465 participants with at least 1 CSF measurement in the DIAN cohort were available from the data freeze 12 (last data from June 30, 2017). Each participant was a member of a pedigree with a known mutation for DIAD. Genotyping was performed to determine the genetic status for each participant at risk; the presence or absence of a DIAD mutation was determined using polymerase chain reaction–based amplification of the appropriate exon followed by Sanger sequencing methods on a genetic analyzer (ABI 3130xl; ThermoFisher Scientific).^21^

Participants were divided into 3 groups according to the presence of a mutation and their Clinical Dementia Rating (CDR)^22^ score at baseline, with a CDR score of 0 indicating no symptoms and a CDR score of 0.5 or higher indicating the presence of symptoms. The participant’s estimated years before expected symptom onset (EYO) was defined as the participant’s age at baseline minus their expected AAO.^6,7,23^ The AAO was calculated according to either the family mutation–specific expected age at dementia onset or parental age at first progressive cognitive decline if the expected AAO for the mutation was unknown. Negative values indicate that an individual is younger than his or her expected AAO.

### Clinical and Neuropsychological Assessments

Participants underwent extensive clinical evaluation, which included family history of AD, personal medical history, and physical and neurological examination. Clinical dementia status was determined with the CDR in accordance with standard protocols and criteria.^22,24^ Clinicians performing the assessments were blinded to mutation status of participants. Full details of participating sites, enrollment, and assessments in DIAN have been published elsewhere.^20^

### Brain Imaging

#### MRI Scanning

Participants underwent volumetric T1-weighted MRI, using the magnetization-prepared rapid acquisition with gradient-echo sequence defined in the ADNI second phase.^25^ Sites used a 3-T scanner and were required to pass regular quality control assessments. Volumetric segmentation and cortical surface reconstruction were performed using FreeSurfer image analyzing software version 5.3 (Harvard Medical School),^26^ and subcortical volumes were corrected for intracranial volume using a regression approach.^27^ Cortical thickness and volume measures were averaged across hemispheres because there were no a priori laterality predictions. Because the focus of our study was to assess the association between longitudinal CSF tTau and pTau181 levels with atrophy rate, a limited number of regions of interest (ROIs) reflecting brain atrophy patterns across AD stages were included. We followed an ROIs approach to include posterior areas with earlier tau deposition (eg, hippocampus, entorhinal cortex, parahippocampus, posterior cingulate gyrus, precuneus gyrus, and supramarginal gyrus) and atrophy vs anterior areas with later tau deposition (eg, superior frontal gyrus, orbital frontal gyrus, and midfrontal gyrus) and atrophy. The ROIs were defined according to areas of early vs late neurodegeneration and tau deposition in the literature and our previous work.^3,14,28,29,30,31^

#### Biochemical Analysis

The CSF was collected in the morning under fasting conditions by lumbar puncture and immediately placed on ice. Samples were shipped on dry ice to the DIAN biomarker core laboratory. We have previously reported a longitudinal decrease in CSF tTau and pTau181 levels using commercially available immunoassays.^14^ To also test whether those findings were specific to the method of measurement, here we used a fully automated, high-performance electroluminescence immunoassay (Elecsys; Roche Diagnostics) of tTau and pTau181 in the laboratory of Leslie Shaw, PhD, in the ADNI Biomarker Core at the University of Pennsylvania according to the kit manufacturer’s instructions and as described in previous studies.^32,33,34^ Analyses were performed in a series of runs, with each sample run 1 time for each of the tau analytes, over the period of October 18, 2017, through November 9, 2017; acceptance criteria as documented according to the Roche Protocol in the University of Pennsylvania ADNI Biomarker Laboratory were followed. In each of the 14 days of performing analyses, the manufacturer’s quality control results were within stated limits to meet acceptance criteria for precision and accuracy. There were 2 runs each day of approximately 40 samples per run. Precision performance was documented according to analysis of pristine aliquots of a CSF pool in each of the 28 runs for each of the biomarkers. The percentage coefficients of variation for this CSF quality control pool were less than 2%.

### Statistical Analysis

 Data analysis was performed in June 2019. Baseline characteristics of the participants are summarized as mean (SD) for continuous variables and number (column percentage) for categorical variables. *P* values for comparing the difference between MCs and noncarriers (NCs) were obtained using generalized linear mixed-effects models with random intercepts for family clusters to take into account the associations between participants within the same family; the tests were 2-sided. The annualized rate of change over the longitudinal follow-up period was estimated for each participant separately using linear regression and then plotted against baseline EYO to evaluate the trajectories of the biomarker changes over EYO. To visualize the differences in the rates of change of different biomarkers as a function of EYO, locally estimated scatterplot smoothing curves of the standardized difference of individual annual rates of change for MCs for different biomarkers were plotted against baseline EYO. A linear or linear spline mixed-effects model for each biomarker (depending on the curve fit) was then used to estimate and test the rates of change at each integer EYO point. The fixed effects in the models included mutation group (MC or NC), baseline EYO, time since baseline, and all possible 2-way or 3-way interactions. Sex, years of education, and apolipoprotein ε4 allele status were considered as covariates, but only significant effects were retained in the models. Random effects included in the models are random intercepts for family clusters, individual random intercept, and random slope. An unstructured covariance matrix was used to account for the within-participant association due to repeated measures. Further details for the linear and linear spline mixed-effects models can be found in the article by McDade et al.^14^

Associations between the individual annual rates of change of tTau and pTau181 concentrations and the annualized rates of brain atrophy were evaluated for MCs with symptoms (CDR score, >0) and MCs without symptoms (CDR score, 0) separately, using bivariate linear mixed-effects models with random intercepts for family clusters.^35,36^ Statistical analyses were conducted with the PROC MIXED and PROC NLMIX procedures in SAS statistical software version 9.4 (SAS Institute Inc). A *P* < .05 was considered to be statistically significant. For CSF biomarkers (145 NCs and 235 MCs), the effect size that could be detected with 80% power was 0.3. For MRI biomarkers (177 NCs and 270 MCs), the effect size that could be detected with 80% power was 0.27.

## Results

We analyzed data from 465 participants, including 283 MCs (183 [65%] without symptoms and 100 with symptoms) and 182 NCs (Table 1). The mean (SD) age of the cohort was 37.8 (11.3) years, and 262 (56.3%) were women. Of the MCs, 213 (75.3%) had presenilin 1 mutations, 22 (7.8%) had presenilin 2 mutations, and 48 (16.9%) had amyloid precursor protein mutations. There were no differences between MCs and NCs in terms of age (mean [SD] age, 37.8 [10.8] vs 37.9 [11.7] years), sex (55.1% vs 58.2% female), educational level (mean [SD] years, 14.3 [3.0] vs 14.8 [2.9]), or the presence of at least 1 apolipoprotein ε4 allele (29.7% vs 30.8%). The EYO for the entire cohort ranged from 38.2 years before the parental AAO to 22.6 years after the parental AAO; however, to reduce the risk of identifying individual participants at the extremes of the EYO range, we show only the EYO interval of −25 to 10 years. The NCs had no or very little evidence of AD pathologic abnormalities and almost all (171 [94.0%]) had normal cognition. At baseline, 183 MCs (64.7%) did not have symptoms (CDR score, 0). The CDR scores of the MCs with symptoms ranged from 0.5 (very mild) to 3 (severe) (Table 1). Two or more longitudinal CSF and MRI assessments were available for 160 and 247 participants, respectively (Table 1 and eTable 1 in the Supplement) with a mean (SD) follow-up of 2.7 (1.5) years.

**Table 1.** Clinical, Cognitive, Imaging, and Biochemical Characteristics of Study Participants at Baseline^a^

Clinical and Baseline Biomarker Characteristics	Mean (SD)		*P* Value
	Noncarriers (n = 182)	Mutation Carriers (n = 283)	
Age, y	37.9 (11.7)	37.8 (10.8)	.80
Female, No. (%)	106 (58.2)	156 (55.1)	.50
Education, y	14.8 (2.9)	14.3 (3.0)	.70
Mini-Mental State Examination score	29.0 (1.2)	26.9 (5.1)	<.001
Clinical Dementia Rating score, No. (%)			
0	171 (94.0)	183 (64.7)	<.001
>0	11 (6.0)	100 (35.3)	
Apolipoprotein ε4 allele carrier, No. (%)	56 (30.8)	84 (29.7)	.80
Estimated years before onset, y	−9.7 (12.2)	−8.2 (11.0)	.20
Length of follow-up, y	3.4 (1.6)	2.9 (1.5)	.03
No. of longitudinal cerebrospinal fluid assessments, No. (%)^b^	55 (37.9)	105 (42.9)	
2	38 (69.1)	69 (65.7)	.24
3	12 (21.8)	25 (23.8)	
≥4	5 (9.1)	11 (10.5)	
Aβ42 level, pg/mL	1385 (471)	961 (623)	<.001
Ratio of Aβ42 to Aβ40	0.09 (0.01)	0.06 (0.03)	<.001
Total tau level, pg/mL	171.6 (58.5)	287.9 (159.1)	<.001
Phosphorylated tau 181 level, pg/mL	14.2 (5.3)	30.8 (22.7)	<.001
Ratio of phosphorylated tau 181 to Aβ42	0.0109 (0.0057)	0.0518 (0.0534)	<.001
No. of longitudinal MRI assessments, No. (%)^c^	89 (50.3)	158 (58.5)	
2	63 (70.8)	96 (60.8)	.09
3	18 (20.2)	47 (29.7)	
≥4	8 (9.0)	15 (9.5)	
Hippocampal volume, mm^3^	8798 (809)	8363 (1302)	<.001
Thickness, mm			
Entorhinal cortical	3.50 (0.30)	3.43 (0.36)	.04
Precuneus	2.38 (0.13)	2.28 (0.22)	<.001
Superior frontal	2.66 (0.13)	2.62 (0.17)	.02

Abbreviation: MRI, magnetic resonance imaging.

^a^ Continuous measures are presented as the mean (SD). For each type of measure, the number of individuals with at least 1 measurement is provided. The significance of the difference between the mutation carriers and noncarriers was calculated by linear mixed-effects models with random intercepts for familial clusters.

^b^ A total of 160 participants underwent longitudinal cerebrospinal fluid assessments.

^c^ A total of 247 participants underwent longitudinal MRI assessments.

### Longitudinal Change in CSF tTau and pTau181 Levels

**Figure 1.**
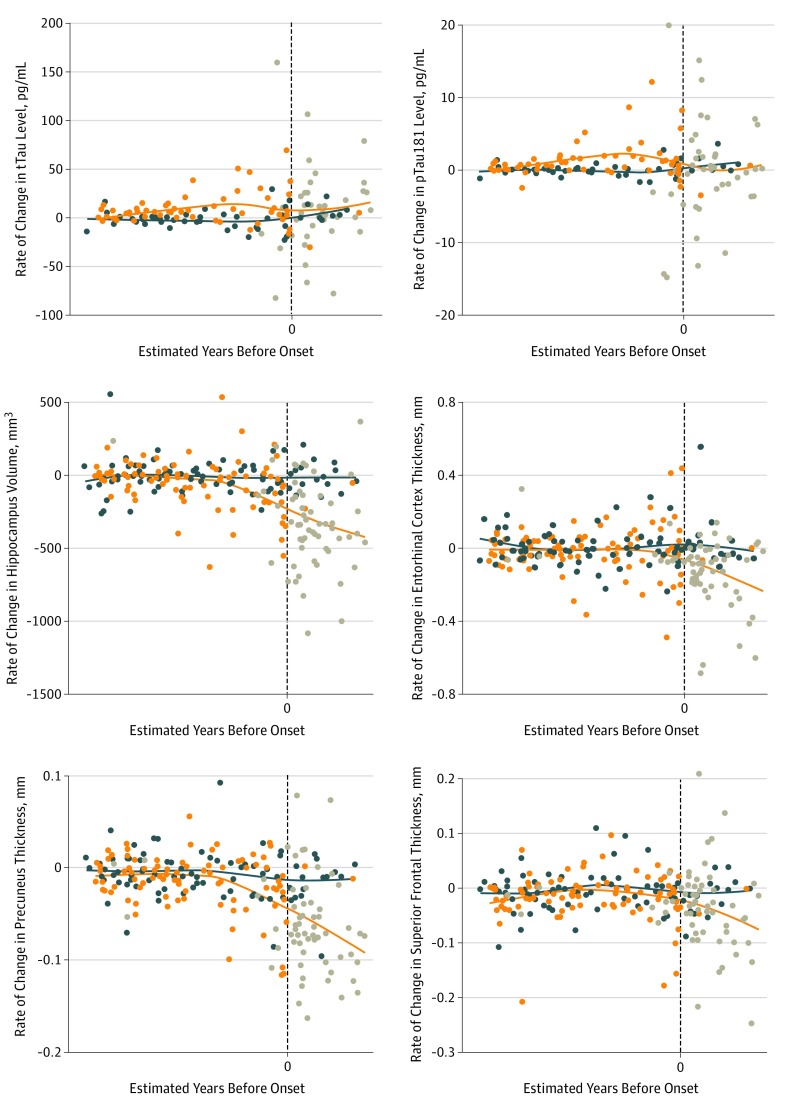
Individual Annual Rate of Change of Cerebrospinal Fluid Levels of Total Tau (tTau) and Phosphorylated Tau 181 (pTau181) and Brain Structures by Magnetic Resonance Imaging as a Function of Baseline Estimated Years Before Onset in Mutation Carriers and Noncarriers Values for noncarriers (blue dots), mutation carriers without symptoms (orange dots), and mutation carriers with symptoms (gray dots) are shown. Blue and orange lines are locally estimated scatterplot smoothing curves for mutation carriers and noncarriers, respectively. To maintain participant and investigator blinding to mutation status when reporting individual data points, specific estimated years before onset are not shown (vertical dashed line indicates year 0). Individual annual rate of change over the longitudinal follow-up was estimated for each participant separately using linear regressions.

Previous analyses^14,17^ of this cohort have found that CSF tTau and pTau181 levels were increased in MCs 15 years before expected symptom onset (EYO = −15). We also examined the longitudinal change in CSF tTau and pTau181 levels in terms of annual rate of change across the EYO (Figure 1 and Table 2). Rates of change for NCs were not significantly different from 0. For MCs, the annual rates of change for CSF tTau and pTau181 became significantly different from 0 near EYO −10 (mean [SE] rates of change, 5.5 [2.8] for tTau [*P* = .05] and 0.7 [0.3] for pTau 181 [*P* = .04]) and EYO −15 (mean [SE] rates of change, 5.4 [3.9] for tTau [*P* = .17] and 1.1 [0.5] for pTau181 [*P* = .03]), respectively (Table 2). Importantly, the longitudinal rates of change of CSF tTau and pTau181 levels depended on where the participant fell with respect to their EYO, and the pattern of change over the AD course was different for tTau and pTau181. Specifically, the positive rate of change of tTau gradually increased until EYO −10 and then remained constant after symptom onset (mean [SE] rate of change, 5.6 [2.3] for EYO 0, 5.6 [3.2] for EYO 5, and 5.7 [4.4] for EYO 10). In contrast, the positive rate of change of pTau181 increased in those at early stages of the disease, starting at EYO −15 until EYO −5 (mean [SE], 0.4 [0.3]), followed by a positive but decreasing rate of change at year 0 (mean [SE] 0.1 [0.3]) and then negative rates of change at EYO 5 (mean [SE], −0.3 [0.4]) and EYO 10 (mean [SE], −0.6 [0.6]) (Table 2), resulting in overall lower levels of pTau181 at later stages of disease.

**Table 2.** Estimated Annual Rate of Change of Cerebrospinal Fluid Levels of tTau and pTau181 and Structural Magnetic Resonance Imaging (Regions of Interest) in Mutation Carriers by Estimated Years Before Onset^a^

Estimated Time Before Onset, y	tTau		pTau181		Entorhinal Thickness		Hippocampal Volume		Precuneus Thickness		Superior Frontal Thickness	
	Rate of Change, Mean (SE)	*P* Value	Rate of Change, Mean (SE)	*P* Value	Rate of Change, Mean (SE)	*P* Value	Rate of Change, Mean (SE)	*P* Value	Rate of Change, Mean (SE)	*P* Value	Rate of Change, Mean (SE)	*P* Value
−25	0.3 (3.8)	.94	0.1 (0.5)	.88	0.011 (0.012)	.33	5.6 (29.0)	.85	−0.004 (0.004)	.37	−0.006 (0.006)	.32
−20	2.9 (2.7)	.28	0.6 (0.3)	.09	−0.002 (0.009)	.83	−23.9 (21.7)	.27	−0.008 (0.003)	.01	−0.008 (0.005)	.09
−15	5.4 (3.9)	.17	1.1 (0.5)	.03	−0.015 (0.007)	.04	−53.5 (17.9)	.004	−0.012 (0.003)	<.001	−0.010 (0.004)	.02
−10	5.5 (2.8)	.05	0.7 (0.3)	.04	−0.028 (0.009)	.001	−83.0 (19.9)	<.001	−0.016 (0.004)	<.001	−0.011 (0.005)	.02
−5	5.5 (2.1)	.01	0.4 (0.3)	.15	−0.041 (0.012)	<.001	−112.5 (26.4)	<.001	−0.020 (0.005)	<.001	−0.013 (0.006)	.04
0	5.6 (2.3)	.02	0.1 (0.3)	.83	−0.066 (0.010)	<.001	−232.9 (20.9)	<.001	−0.048 (0.003)	<.001	−0.022 (0.005)	<.001
5	5.6 (3.2)	.08	−0.3 (0.4)	.51	−0.106 (0.018)	<.001	−413.9 (34.4)	<.001	−0.075 (0.005)	<.001	−0.036 (0.008)	<.001
10	5.7 (4.4)	.20	−0.6 (0.6)	.28	−0.147 (0.033)	<.001	−594.9 (61.6)	<.001	−0.103 (0.009)	<.001	−0.050 (0.014)	<.001

Abbreviations: pTau181, phosphorylated tau 181; tTau, total tau.

^a^ *P* values were for testing whether the rate of change is significantly different from 0.

### Longitudinal Change in Brain Volumes

The A/T/N framework uses CSF tTau levels and/or structural MRI measurements as a marker of neurodegeneration, so we sought to determine whether CSF tTau could be used as a suitable biomarker to track MRI atrophy rate. For MRI measurements in MCs, the individual annual rate of change in precuneus thickness started to show a difference from 0 approximately 20 years before expected symptom onset (mean [SE] rate of change, −0.008 [0.003]), followed by rate of change in hippocampal volume (mean [SE] rate of change, −53.5 [17.9]), entorhinal thickness (mean [SE] rate of change, −0.015 [0.007]), and superior-frontal thickness (mean [SE] rate of change, −0.010 [0.004]) around EYO −15. The longitudinal rate of change for all measured brain ROI increased 5 years before symptoms appeared (EYO −5) and followed a similar trend through the disease (Table 2 and Figure 1). For all ROIs included in the analysis, the rate of change followed similar trends.

**Figure 2.**
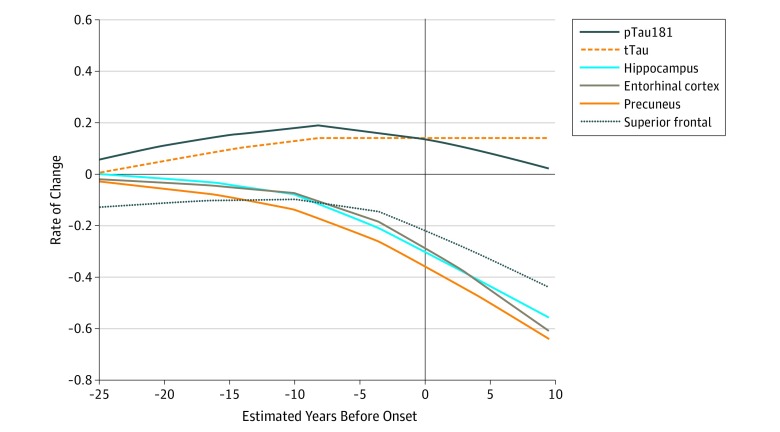
Comparison of the Rates of Change of Cerebrospinal Fluid Levels of Total Tau (tTau) and Phosphorylated Tau 181 (pTau181) and Brain Structures by Magnetic Resonance Imaging in Mutation Carriers as a Function of Estimated Years Before Onset Lines are locally estimated scatterplot smoothing curves for the standardized difference in individual annual rate of change. Values represent the estimated rate of change based on the longitudinal data of the mutation carriers standardized by the mean and SD of the baseline value of noncarriers .

To visually compare the differences in the trajectories of the rates of change of CSF tTau and pTau181 levels and structural MRI measures, locally estimated scatterplot smoothing curves were constructed for the standardized rate of change as a function of baseline EYO (Figure 2). Notably, after EYO 0, the rate of change became more negative (structures atrophied more quickly), compared with CSF tTau levels, which continued to have a stable positive rate of change.

### Association Between CSF and MRI Measures of Neurodegeneration

**Figure 3.**
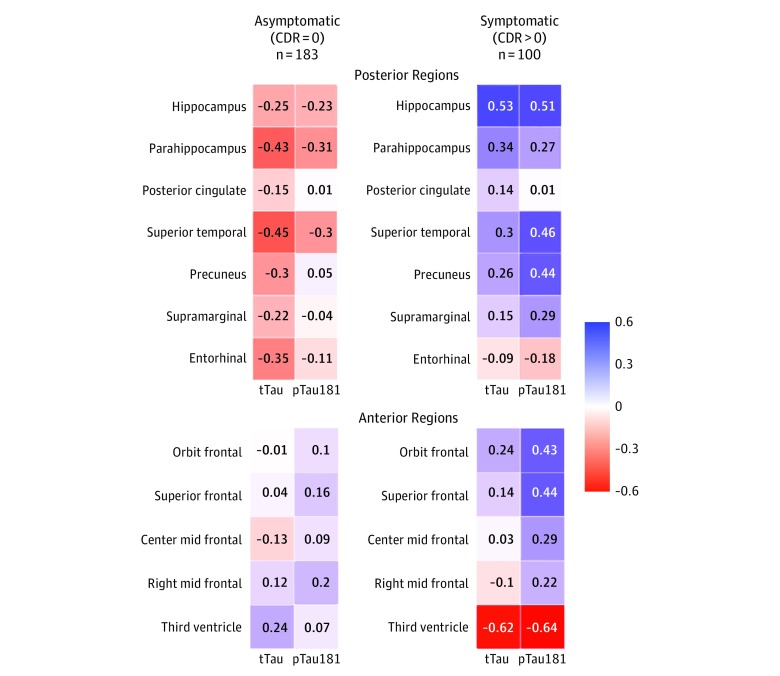
Correlation Coefficient Maps for the Individual Annual Rate of Change of Cerebrospinal Fluid Levels of Total Tau (tTau) and Phosphorylated Tau 181 (pTau181) and Brain Structures by Clinical Dementia Rating (CDR) Group and Early (Posterior) and Late (Anterior) Brain Regions The color-coding and the number in each cell represent the estimated correlation coefficient between the individual annual rate of change of cerebrospinal fluid tTau and pTau181 and cortical thickness.

We found differences in nearly all posterior and limbic or paralimibic regions for associations in rates of change with tTau and pTau181 according to stage of disease when comparing MCs without symptoms vs those with symptoms (eTable 2 and eTable 3 in the Supplement). Figure 3 shows patterns of correlation coefficients between the rates of change of CSF tTau and pTau181 and brain structure stratified by the absence or presence of symptoms (CDR score of 0 vs CDR score >0) and by posterior (early atrophy) and anterior (later atrophy) cortical or subcortical regions. In individuals without symptoms (CDR score of 0), the rates of change of CSF tTau and pTau181 were inversely correlated with most brain structure measures, where higher CSF tTau levels were associated with smaller cortical thickness. Generally, there were higher correlation coefficients for CSF tTau in the asymptomatic phase and mostly for the posterior neocortical and allocortical (limbic) regions. However, after symptom onset, the correlation between CSF tTau and pTau181 and brain structures changed. The intensity of neuronal damage as measured by brain atrophy continued at an increasing rate, whereas the rate of change of CSF tTau levels remained at a somewhat constant rate and the rate of change of CSF pTau181 actually switched from positive to negative. These results suggest that CSF tTau and structural brain measures have distinct patterns later in the disease course.

## Discussion

The use of biomarkers has become an essential component of AD research^11,37^ and therapeutic trials. The new framework from the National Institute on Aging and Alzheimer Association^11^ capitalizes on the use of biomarkers for early identification of AD, which has substantial implications for early treatment and trial enrollment. However, little work has been done regarding comparisons of longitudinal biomarker trajectories that are currently proposed to represent similar aspects of disease (eg, CSF tTau and MRI are both proposed as markers of neurodegeneration).^38^

In this study, on the basis of longitudinal data from the DIAN study,^20^ we evaluated the trajectories of CSF tTau and pTau181 over the course of AD. Notably, we included a bigger sample and used newer CSF tTau and pTau181 values generated with a fully automated, high-performance electroluminescence immunoassay. We compared the trajectories of CSF tTau and pTau181 with the atrophy of brain structures as measured by MRI. First, consistent with previous reports,^14,39^ our study found mean concentrations of CSF tTau and pTau181 to be higher in MCs from the early stage of AD, supporting the use of CSF tau as a marker of AD risk and progression. Second, we found that the positive rate of change of CSF tTau remained constant after EYO −10, whereas CSF pTau181 had a positive rate of change early in the disease course, which then reversed and became negative later in the disease. This indicates that our previous results^13,14^ were not likely to be an artifact of the measurement used because we used a different method for this study. Third, the associations identified between the rates of change of CSF tTau and pTau181 with brain atrophy do not support the assumption that CSF tTau changes follow a pattern similar to that of structural brain changes. Our findings indicate that neither CSF tTau nor pTau181 has the same pattern of change as brain measures and should be considered as associated but distinct biomarkers in AD. In addition, these findings indicate that, within the current biomarker classification, tTau is an important marker of AD but may not be the ideal marker of neurodegeneration.

Recent studies^40,41^ have suggested that in the presence of amyloid pathologic abnormalities, more CSF tTau and pTau181 is released. Moreover, increased tau in CSF seems to be dependent of amyloid deposition and occurs in the absence of tau brain pathologic abnormalities.^42^

Early disease stages may also be characterized by higher cellular stress^43,44^ and inflammation, with higher levels of tTau and pTau181 in CSF representing a response.^45^ However, if CSF tTau directly reflected neurodegeneration, it would be expected that the rate of change of CSF tTau would increase in concert with brain atrophy during the period of maximal rate of atrophy (EYO >0). It is possible that during disease progression and neurodegeneration, the loss of neuronal cells results in less neuronal substrate to produce tau. Although this might account for some of the slowing in longitudinal changes in CSF tTau that we observed, it is unlikely that the degree of neurodegeneration is sufficient to fully explain our findings. Early elevations may also be associated with acute neuronal membrane damage, whereas apparent later reductions reflect the death of a smaller number of neurons that remain. Acute neuronal injury may be associated with a stronger inflammatory response at early stages of the disease.^46^

The present findings challenge some previous assumptions about AD progression and its association with both pTau181 and tTau. Contrary to the idea that tTau and pTau181 levels continue to increase with greater neurodegeneration and the spread of NFT,^47^ we found evidence of a decrease in the rate of change, arguing against the use of these measures as a reflection of a passive release from neuronal death and NFT. These data support the relevance of CSF tTau and pTau181 as markers of amyloid deposition and accompanying changes (eg, inflammation and neuronal membrane damage)^11^; however, the complex rates of change identified here and in our previous work suggest that using them as measures of therapeutic response requires further investigation, because levels vary as a function of where an individual is in the neuropathological cascade. The apparent disconnect between CSF tTau and MRI measures may reflect the fact that they are measuring different stages of the neurodegenerative process, with CSF tTau accounting for the active phase of neuron injury and damage, and MRI measuring the subsequent structural sequelae of the active death process.

Our findings are consistent with previous studies^15,38,48,49,50^ of sporadic late-onset AD. Seppälä et al,^48^ in a longitudinal study in Finland, described higher levels of pTau181 level in mild cognitive impairment stages when compared with AD dementia, whereas levels of pTau181 decreased over a period of about 3 years. Similarly, Toledo et al^38^ reported a decrease in tTau in those at the dementia stage from the ADNI cohort. More recently, in a similar analysis of ADNI participants with longitudinal follow-up and using similar methods to determine CSF tTau and pTau181 levels (ie, automated electroluminescence immunoassay), Sutphen et al^15^ reported that tTau and pTau181 showed consistent increases in the amyloid-positive participants with normal cognition and those with mild cognitive impairment, whereas pTau181 decreased substantially in those with AD dementia.

This study has potential implications for AD trials using tau-based therapies and other putative disease-modifying therapies. First, during trial design, the active group and the placebo group will have to be randomized by disease stage (according to disease severity measured using biomarkers and the severity of cognitive impairment), because minor differences in the neurodegeneration cascade stage might translate into major differences in biomarker trajectories, and as a result, might be misinterpreted as a treatment effect. Second, one must consider how to interpret changes in biomarkers during clinical trials readout; in other words, one must consider how a successful treatment would be expected to affect CSF tau levels or rate of change. This study, along with recent work assessing neurofilament light chains,^51,52^ suggests that neurofilament light chains may be an advantageous marker of neurodegeneration in therapeutic trials of AD.^52,53^

### Limitations

The present study is not without limitations. First, our results are dependent on the accuracy of the DIAN EYO and, like any measure, it is subject to error (ie, symptom onset could be a few years earlier or later than expected [randomly]). Second, we recognize that DIAD and late-onset AD are similar but not identical. Sporadic AD disease occurs later in life, and individuals often exhibit a wide range of comorbidities, including vascular disease and the presence of transactive response DNA-binding protein of 43 kDa.^54,55,56,57^ These differences may be associated with biomarker profiles or natural progression, so the total generalizability of the present findings to late-onset AD will have to be confirmed in future cohorts. Nevertheless, the results highlight the importance of gathering longitudinal data to refine current biomarker models.

## Conclusions

The results of this study support the A/T/N framework, whereby increases in CSF levels of tTau and pTau181 are robust diagnostic markers of pathologic abnormalities and neuronal injury in early Alzheimer disease. However, our data suggest that levels of CSF tTau and pTau181 diverge from atrophy-based measures of neurodegeneration later in the disease. This is an important result to consider in clinical trials targeting tau brain pathologic abnormalities. Future studies should explore the longitudinal rate of change of CSF pTau181 levels and positron emission tomography of tau as markers of tau brain pathologic abnormalities.

## Notes

Supplement. **eTable 1.** Number of Participants With Longitudinal CSF Biomarkers and MRI Assessments **eTable 2.** Associations Between CSF tTau and MRI Regions by Disease Stage (Asymptomatic vs Symptomatic) **eTable 3. **Associations Between CSF pTau181 and MRI Regions by Disease Stage (Asymptomatic vs Symptomatic)
