# Supplementary material for: Association of Longitudinal Changes in Cerebrospinal Fluid Total Tau and Phosphorylated Tau 181 and Brain Atrophy With Disease Progression in Patients With Alzheimer Disease
Source: JAMA Netw Open. 2019 Dec 11;2(12):e1917126. doi: 10.1001/jamanetworkopen.2019.17126 (PMC6991202; doi:10.1001/jamanetworkopen.2019.17126)
Supplement: Supplement. — eTable 1. Number of Participants With Longitudinal CSF Biomarkers and MRI Assessments eTable 2. Associations Between CSF tTau and MRI Regions by Disease Stage (Asymptomatic vs Symptomatic) eTable 3. Associations Between CSF pTau181 and MRI Regions by Disease Stage (Asymptomatic vs Symptomatic) [file jamanetwopen-2-e1917126-s001.pdf]

Supplementary Online Content

Llibre-Guerra JJ, Li Y, Schindler SE, et al. Association of longitudinal changes in cerebrospinal fluid total tau and phosphorylated tau 181 and brain atrophy with disease progression in patients with Alzheimer disease. *JAMA Netw Open*. 2019;2(12):e1917126. doi:10.1001/jamanetworkopen.2019.17126

eTable 1. Number of Participants With Longitudinal CSF Biomarkers and MRI Assessments

eTable 2. Associations Between CSF tTau and MRI Regions by Disease Stage (Asymptomatic vs Symptomatic)

eTable 3. Associations Between CSF pTau181 and MRI Regions by Disease Stage (Asymptomatic vs Symptomatic)

This supplementary material has been provided by the authors to give readers additional information about their work.

© 2019 Llibre-Guerra JJ et al. *JAMA Network Open.*

eTable 1. Number of Participants With Longitudinal CSF Biomarkers and MRI Assessments

	CSF biomarker			MRI		
# of visits	**NC**	**MC**	**Total**	**NC**	**MC**	**Total**
1	90	130	220	88	112	200
2	38	69	107	63	96	159
3	12	25	37	18	47	65
4	4	5	9	4	7	11
5	1	5	6	3	5	8
6	0	1	1	1	3	4
Total	145	235	380	177	270	447

NC=non-carriers, MC=mutation carriers

© 2019 Llibre-Guerra JJ et al. *JAMA Network Open.*

eTable 2. Associations Between CSF tTau and MRI Regions by Disease Stage (Asymptomatic vs Symptomatic)

tTau	Asymptomatic MC (aMC)		Symptomatic MC (sMC)		p value for difference in r between aMC and sMC
	r	p value	r	p value	
Hippocampus	-0.25	0.03	0.53	0.04	<0.0001
Parahippocampus	-0.43	< 0.001	0.34	0.11	<0.0001
Post.Cing	-0.15	0.26	0.14	0.57	0.16
Sup. Temp	-0.45	<0.001	0.30	0.13	0.0001
Precuneus	-0.30	0.03	0.26	0.26	0.005
Supramarginal	-0.22	0.17	0.15	0.43	0.07
Entorhinal	-0.35	0.15	-0.09	0.66	0.19
Orbit Frontal	-0.01	0.97	0.24	0.18	0.22
Sup. Frontal	0.04	0.81	0.14	0.48	0.63
C.Mid.Front	-0.13	0.4	0.03	0.89	0.44
R.Mid.Frontal	0.12	0.47	-0.10	0.62	0.29
Third.Vent	0.24	0.04	-0.62	<0.001	<0.0001

© 2019 Llibre-Guerra JJ et al. *JAMA Network Open.*

eTable 3. Associations Between CSF pTau181 and MRI Regions by Disease Stage (Asymptomatic vs Symptomatic)

pTau181	Asymptomatic MC (aMC)		Symptomatic MC (sMC)		p value for difference in r between aMC and sMC
	r	p value	r	p value	
Hippocampus	-0.23	0.05	0.51	0.01	<0.0001
Parahippocampus	-0.31	0.005	0.27	0.12	<0.0001
Post.Cing	0.01	0.92	0.01	0.96	1
Sup. Temp	-0.30	0.01	0.46	0.01	0.0001
Precuneus	0.05	0.74	0.44	0.02	0.04
Supramarginal	-0.04	0.75	0.29	0.09	0.1
Entorhinal	-0.11	0.39	-0.18	0.36	0.73
Orbit Frontal	0.10	0.44	0.43	0.01	0.08
Sup. Frontal	0.16	0.24	0.44	0.01	0.13
C.Mid.Front	0.09	0.46	0.29	0.10	0.32
R.Mid.Frontal	0.20	0.14	0.22	0.26	0.92
Third.Vent	0.07	0.58	-0.64	<0.001	<0.0001

© 2019 Llibre-Guerra JJ et al. *JAMA Network Open.*
